# Patient with AL amyloidosis with recurrent syncope as the first symptom: case report and literature review

**DOI:** 10.3389/fonc.2026.1744590

**Published:** 2026-03-20

**Authors:** Lingling Hu, Lulu Luo, Nan Yao, Jie Mi, Yuexian Zhang, Ming Sun, Huaping Wang, Yanling Xu, Wei Wang, Jiong Hu, Hongbin Lu

**Affiliations:** 1 Department of Hematology, Wuxi Branch of Ruijin Hospital Shanghai Jiao Tong University School of Medicine, Wuxi, China; 2 Department of Radiation Oncology, The Affiliated Wuxi People’s Hospital of Nanjing Medical University, Wuxi, China; 3 Shanghai Institute of Hematology, Blood and Marrow Transplantation Center, Collaborative Innovation Center of Hematology, Department of Hematology, Ruijin Hospital, Shanghai Jiao Tong University School of Medicine, Shanghai, China

**Keywords:** autonomic nervous system, daratumumab, hypotension, light chain amyloidosis, syncope

## Abstract

**Background:**

Immunoglobulin light-chain (AL) amyloidosis is a rare systemic disease caused by the extracellular deposition of misfolded light chains. Nonspecific manifestations, especially those dominated by severe autonomic neuropathy, frequently delay diagnosis.

**Methods and results:**

A 68-year-old woman was admitted after five months of recurrent syncope. The clinical picture was dominated by refractory orthostatic hypotension, nausea and vomiting alternating with diarrhea and constipation, stocking-glove paresthesias, and proteinuria. Bone marrow examination showed the presence of clonal plasma cells with positive M protein, which was confirmed by the serum free light chain test. AL amyloidosis was diagnosed based on positive Congo red staining in renal biopsy specimens. The patient was enrolled in a daratumumab-based protocol combined with daratumumab, bortezomib, cyclophosphamide, and dexamethasone. After three cycles, complete hematological remission was achieved, and the autonomic nerve function improved gradually over the subsequent six months.

**Conclusion:**

This case and literature review suggest that AL amyloidosis should be considered whenever a patient presents with multisystem dysfunction dominated by autonomic nerve dysfunction. Although such presentations are associated with poor prognosis, prompt initiation of daratumumab-based therapy can produce rapid hematologic remission and clinically meaningful improvement.

## 1 Introduction

Based on histopathology, AL amyloidosis can be subclassified by the deposited monoclonal light chain into λ-restricted (approximately 85% of cases) or κ-restricted forms (1). More than 30 precursor proteins have been implicated in systemic amyloid disease, and this number continues to rise as mass-spectrometric classification becomes routine (2). AL amyloidosis is almost always underpinned by plasma cell dyscrasia, most commonly monoclonal gammopathy of undetermined significance (MGUS) or overt multiple myeloma, and preferentially involves the kidneys, liver, and heart. Diagnosis is based on the demonstration of Congo red–positive amorphous deposits lining the basement membranes. Neurological involvement, manifesting as peripheral motor, sensory, or autonomic neuropathy, occurs in approximately 15% of patients (3). Deposition within the endoneurial and perivascular spaces leads to mechanical compression and ischemia, producing autonomic failure and neurogenic hypotension.

We report an unusual case of AL amyloidosis that initially presented with recurrent syncope, limb weakness, and refractory hypotension. The diagnostic procedure illustrates the challenge of recognizing amyloidosis when neurological symptoms precede overt organ dysfunction. Prompt treatment with a daratumumab-based regimen resulted in rapid clinical improvement. Our experience underscores the importance of considering amyloidosis in patients with unexplained autonomic instability.

## 2 Case presentation

### 2.1 Patient’s history and physical examination

This is a 68-year-old female patient. In October 2022, the patient experienced intermittent dizziness. By February 2023, the vertigo had intensified and was accompanied by limb weakness severe enough to cause falls; home blood pressure readings were approximately equal to 120/70 mmHg. Neurological examination at that time was unremarkable. Cranial CT revealed chronic ischemic foci adjacent to the lateral ventricles and cerebral atrophy. Lumbar puncture yielded cerebrospinal fluid (CSF) with a total protein level of 769.62 mg/L, a positive Pandy test result, normal glucose and chloride levels, and a negative cryptococcal antigen test result. MRI of the lumbar spine demonstrated multilevel disc bulging and protrusion, spinal canal narrowing, end-plate inflammatory changes, and degenerative disease; cervical MRI showed similar spondylotic changes. She was treated empirically with statins, antiplatelet agents, neurotrophic and vasoactive drugs, antihypertensives, anti-vertiginous medication, and anxiolytics. In March 2023, the patient began experiencing recurrent syncope precipitated by walking, and her dizziness improved only when lying flat. These episodes were accompanied by nausea, vomiting, and progressive numbness in both lower limbs. Orthopedic reassessment attributed the symptoms to lumbar spinal stenosis and herniated discs. Notably, she had lost 10 kg in the preceding year.

Her past medical history included anxiety disorder treated with oral Derivan, well-controlled hypertension on amlodipine besylate (with prior SBP >180 mmHg to approximately 120/60 mmHg), cervical disc protrusion, erosive gastritis, and chronic hepatitis B currently maintained on entecavir. The patient denied any known hereditary diseases in her family.

Physical examination showed the following vital signs: supine BP 138/70 mmHg with unavailable standing BP since the patient collapsed from dizziness within seconds. Additionally, our institution lacks HRV and QSART equipment, preventing us from performing other relevant tests for autonomic nervous function assessment. The patient presented with normal motor strength bilaterally, and electrophysiology confirmed peripheral sensorimotor axonal polyneuropathy with delayed conduction velocities. The systemic survey showed no macroglossia, peripheral edema, or palpable hepatosplenomegaly.

### 2.2 Diagnostic workup

#### 2.2.1 Laboratory findings

Laboratory summary showed normal CBC, coagulation profile, and negative tumor markers with decreased serum albumin (33.0 g/L), creatinine (86 µmol/L), and BNP (56 pg/mL) levels in biochemistry tests. The immunology screening test showed positive ANA (nucleolar pattern 1:320) with negative anti-PCNA and ANCA results. Serum immunofixation demonstrated a monoclonal IgA-λ, and sFLC analysis showed κ 22.2 mg/L, and λ 248.75 mg/L with a κ/λ ratio of 0.09, consistent with a λ-predominant monoclonal process. Urinalysis revealed protein 4+ with occult blood 2 +.

#### 2.2.2 Histopathology and immunohistochemistry

Bone marrow examination showed hypercellular marrow with trilineage (granulocytic, erythroid, and megakaryocytic) hyperplasia, and platelets were present singly and/or in aggregates. The biopsy demonstrated a myeloproliferative pattern with trilineage hyperplasia and a mildly increased plasma cell infiltrate, raising concerns for early plasma cell dyscrasia.

**Figure 1:**
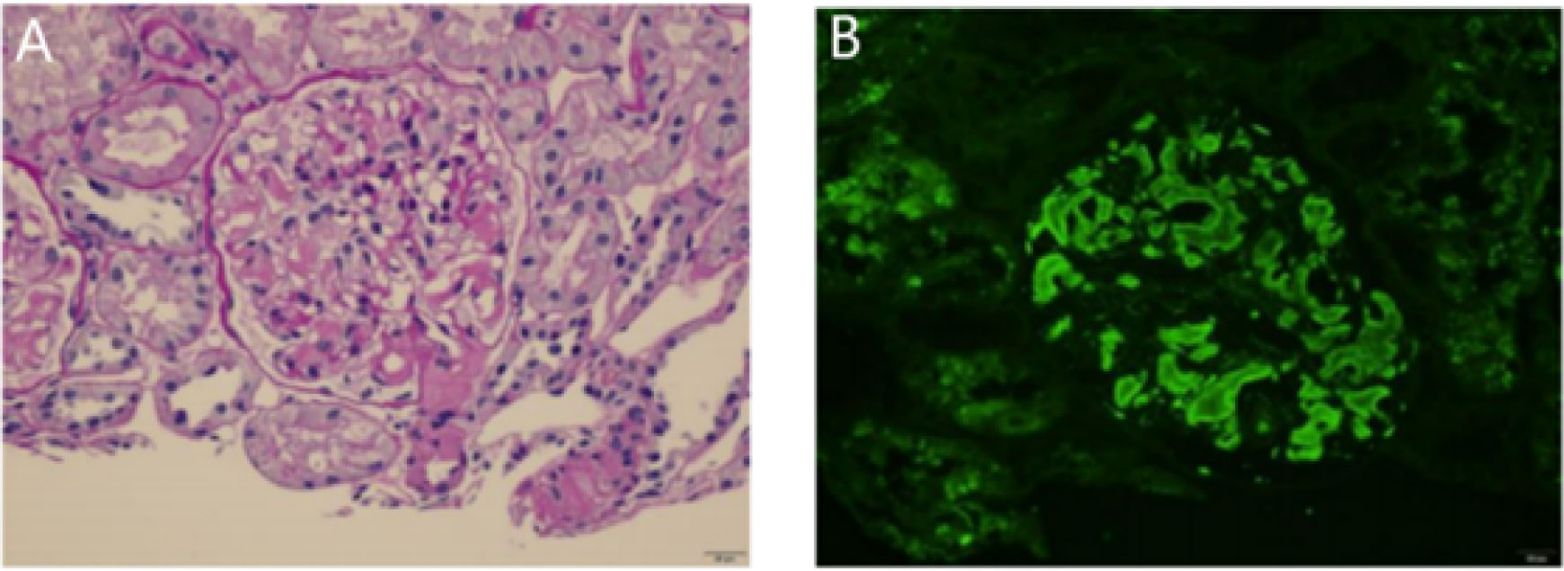
Renal puncture pathology. **(A)** PAS stain. **(B)** light chain λ (×400).

Renal biopsy established the diagnosis: Congo red–positive deposits exhibiting apple-green birefringence under polarized light confirmed renal amyloidosis of the λ light-chain (AL-λ) type, as shown in **Figure 1**.

#### 2.2.3 Imaging examinations

The cranial CT scan was unremarkable. Lumbar spine MRI revealed multilevel degenerative spondylosis and canal stenosis with L3–4 and L4–5 disc bulges, L5–S1 herniation, adjacent vertebral endplate (Modic) changes, a cystic lesion in the S2 canal, simple cysts in the left kidney, and mild lumbar subcutaneous edema. Echocardiogram showed isolated grade I diastolic dysfunction without wall thickening or reduced EF.

#### 2.2.4 Diagnosis and treatment process

The constellation of Congo red–positive λ light-chain renal amyloid, monoclonal IgA-λ on immunofixation, suppressed κ/λ ratio, nephrotic-range proteinuria, orthostatic hypotension with absent standing BP, and sensorimotor axonal polyneuropathy secures the diagnosis of systemic AL (λ) amyloidosis with autonomic, renal, and peripheral nerve involvement. We further evaluated whether the patient’s heart was involved. The patient did not present with clinical manifestations such as chest tightness, dyspnea, jugular venous distention, edema, palpitations, or arrhythmias. The patient’s creatine kinase isoenzymes, myoglobin, and high-sensitivity troponin levels within the normal range. Electrocardiography revealed a sinus rhythm. Echocardiography revealed no thickening of the left or right ventricular walls, interventricular septum, or left or right atria, and a left ventricular ejection fraction of 63%. Cardiac magnetic resonance imaging could not be performed because of the patient’s requirement for continuous bed rest and ongoing dopamine therapy. Based on the above, we assessed the patient’s heart as unaffected.

Coagulation re-check on admission revealed a markedly elevated D-dimer level (10.24 μg/mL). Duplex ultrasound of the lower limbs confirmed bilateral arterial microcalcifications and an acute occlusive thrombus in several left calf interosseous veins. Therapeutic-dose enoxaparin (1 mg/kg s.c. every 12 h) was administered.

**Figure 2:**
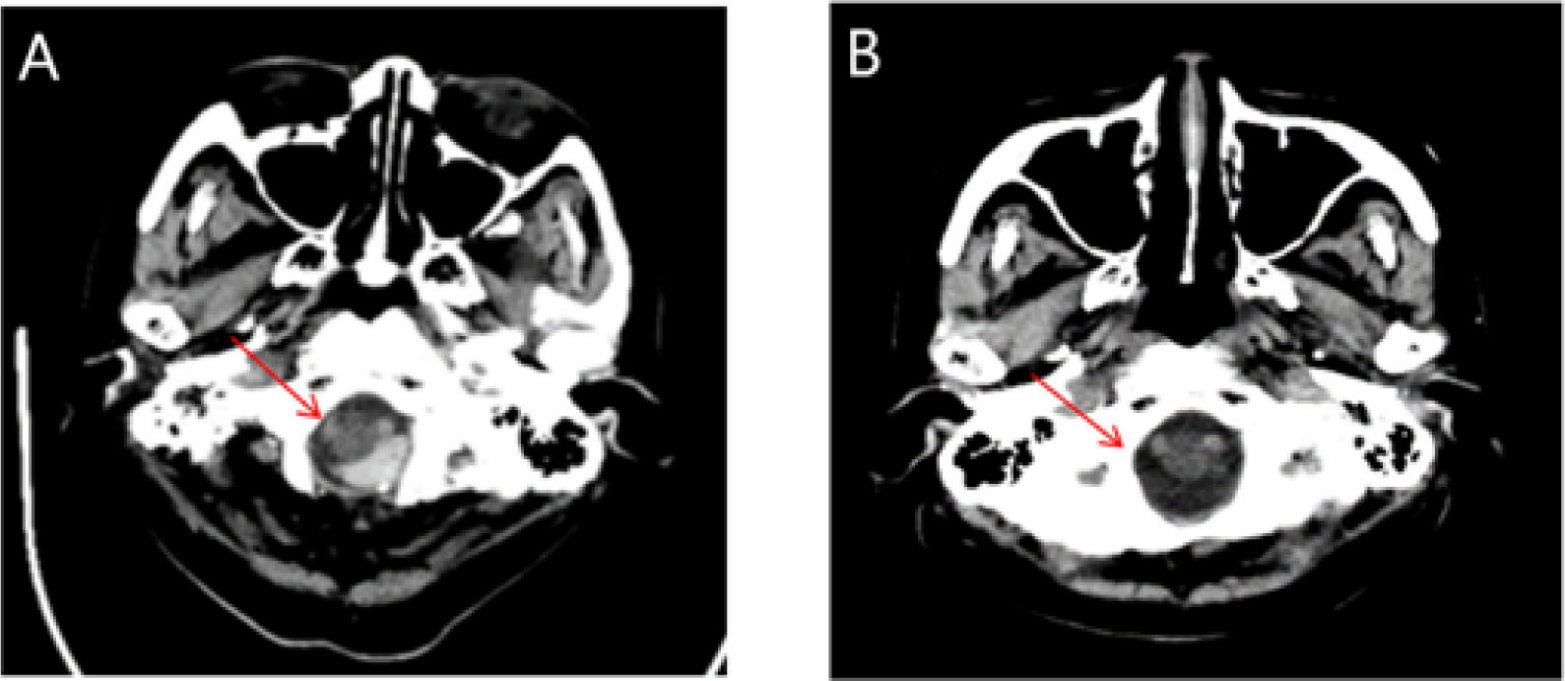
CT images of the patient during and after absorption of intracranial hemorrhage. **(A)** Headache on the day of cranial CT Examination. **(B)** Cranial CT on the 10th day after bleeding treatment.

Chemotherapy was initiated with daratumumab plus VCd (bortezomib–cyclophosphamide–dexamethasone). On cycle-1 day 3, the patient lost consciousness due to severe orthostatic hypotension and struck her head when she attempted to go to the toilet. Emergency cranial CT revealed a posterior medullary hematoma (**Figure 2A**). She remained hypotensive (SBP <80 mmHg) and was managed with continuous dopamine infusion and strict bed rest. Ten days later, a repeat CT showed complete absorption of the hemorrhage (**Figure 2B**), and dopamine was weaned off.

Following recovery from the cerebral hemorrhage, the patient resumed induction therapy with a regimen of daratumumab plus VCd (bortezomib–cyclophosphamide–dexamethasone). The specific dose and schedule were as follows: (Cycle 1) daratumumab 800 mg intravenous (IV) on Day 4; bortezomib 1.5 mg/m² subcutaneous injection(SC) on Days 3, 7, and 18; cyclophosphamide 300 mg/m² IV on Days 3 and 17; dexamethasone 20 mg IV on Days 1 and 18. (Cycle 2) Daratumumab 800 mg IV on Day 1, bortezomib 1.5 mg/m² SC on Day 8, and dexamethasone 20 mg on Days 1 and 8. Owing to the financial constraints of the patient’s family, the dose of daratumumab was reduced. Given the patient’s weakened physical condition, the doses of bortezomib, cyclophosphamide, and dexamethasone were lowered. After completing two cycles of the reduced-dose daratumumab-VCd regimen, the patient’s family refused all further anti-plasma cell therapy due to financial reasons and loss of confidence in the treatment. Throughout therapy, the patient remained profoundly hypotensive and required continuous IV dopamine hydrochloride infusion, with peak daily doses reaching 800 mg. The patient had previously used elastic stockings, received intravenous fluid expansion, and took midodrine hydrochloride without significant therapeutic effects. Subsequently, midodrine hydrochloride was discontinued because of intestinal obstruction and vomiting. Given the absence of specific guidelines for administering vasoactive medications in severe neurogenic hypotensive shock, dopamine was administered for blood pressure elevation according to the Surviving Sepsis Campaign: International Guidelines for Management of Sepsis and Septic Shock 2021 (4). She experienced multiple abrupt syncopal episodes accompanied by non-detectable blood pressure. Consciousness was rapidly restored each time after a brief, rapid IV bolus of dopamine. A trial switch to norepinephrine was attempted but abandoned because of tachycardia.

### 2.3 Treatment response and clinical course

Since the patient and her family members declined further treatment, no further anti-plasma cell disease treatment was administered after June 2023, except for courses of dexamethasone. Following the discontinuation of anti-plasma cell therapy, the serum-free light chain levels remained stable. As of January 2024, although a slight increase was observed (**Table 1**), the response was assessed as Very Good Partial Response, and the 24-hour urinary protein decreased to 27 g/L. Autonomic function improved markedly, and dopamine was discontinued after 9 months of treatment, with normalized orthostatic blood pressure. The urinary retention resolved together with partially diminished lower-limb numbness and subsided intractable nausea and vomiting. The patient resumed independent ambulation, and parenteral nutrition was stopped before discharge. At the last follow-up, the patient remained in good general condition, was fully independent in activities of daily living, and was able to ambulate without assistance.

**Table 1:** Serum free light chain post-treatment change levels.

Dates	March 2023	May 2023	June 2023	October 2023	January 2024
FLC κ (mg/L)	22.2	12.12	13.4	18.1	56.72
FLC λ (mg/L)	248.75	11.08	14	18.7	33.26
κ/λ ratio:	0.09	1.09	0.96	0.97	1.71

FLC, free light chain.

## 3 Literature review

A PubMed search (from inception to May 2025) for “Autonomic Nervous System” AND “Light Chain Amyloidosis” identified six reported cases of systemic AL amyloidosis with clinically overt autonomic dysfunction. The key characteristics of these cases are presented in **Table 2**. The cohort comprised four men and two women aged 56–78 years, with universal autonomic failure. Although peripheral neuropathy was reported in only approximately 15% of unselected AL patients, it was present in all six patients in this study. Five patients died despite supportive care, with only one long-term survivor receiving high-dose corticosteroids. There was one prior report describing recurrent syncope as the sentinel manifestation; however, that patient did not receive disease-directed therapy, highlighting the rarity and poor prognosis of profound autonomic involvement in untreated AL amyloidosis.

**Table 2:** Characteristics of patients with light−chain amyloidosis associated with autonomic symptoms from current case and literature review.

Case number and references	Age/Sex	Site of amyloid deposition	Peripheral nervous system involvement	Mainly characterized	Treatment	Outcome
P1 This case	68/F	Kidneys	YES	Dizziness, Hypotension	Daretuzumab in combination with the VCD	Survived
P2 (5)	67/M	Leptomeningeal, cortical, subcortical arteries as well as in the capillaries, subcutaneous fat	YES	Disorientation, ataxia	Sortezomib	NA
P3 (6)	68/M	Endoneurial blood vessels	YES	Hypotension	Large dose of dexamethasone	Survived
P4 (7)	73/F	Hyperemic gastric antrum pointed out perivascular deposits	YES	Refractory hypotension, diarrhea, dysphagia	Fludrocortisone and midodrine	Death
P5 (8)	56/M	Endomyocardial	YES	Urinary retention, erectile dysfunction, diarrhea, constipation	Transferred to hematology for chemotherapy	NA
P6 (9)	78/F	Bone marrow biopsy, fat aspirate, a lip biopsy	YES	Orthostatic hypotension, diarrhea	Melphalan and dexamethasone	Death
P7 (10)	76/F	gastrointestinal biopsy	NO	Syncope, orthostatic hypotension	Supportive treatments such as pressure enhancement	Request discharge

NA, non-available; F, female; M, male.

## 4 Discussion

Early mortality data from the Mayo Clinic indicate that approximately or equal to 20% of patients with newly diagnosed AL amyloidosis succumb within six months, a figure essentially unchanged over the past four decades and a stark reminder that diagnostic delay remains largely unmitigated (11). According to the 2021 International Myeloma Working Group consensus (12), the diagnosis of light-chain (AL) amyloidosis requires three elements: 1) objective evidence of tissue/organ dysfunction on clinical, laboratory, or imaging tests; 2) tissue biopsy and histopathological analysis demonstrating amyloid deposition with immunohistochemistry or mass spectrometry confirmation that the precursor protein is an immunoglobulin light or heavy-light chain; and 3) demonstration of a circulating monoclonal immunoglobulin or free-light-chain (FLC) abnormality and/or clonal plasma or B-cells in the marrow.

Autonomic neuropathy is one of the most devastating complications of AL. The clinical hallmarks of MSA include severe orthostatic hypotension and gastroparesis with alternating diarrhea and constipation (13). Pathogenetically, misfolded light-chain fibrils infiltrate and compress autonomic nerve fibers within the endoneurium and perineurium, disrupt microvascular perfusion, and ultimately produce irreversible axonal degeneration, culminating in neurogenic orthostatic hypotension and multi-system autonomic failure. The index patient we reported had primary λ-type AL with autonomic neuropathy, marked by significant orthostatic hypotension with repeated syncope and gastroparesis.

Autonomic neuropathy—orthostatic hypotension, gastroparesis, and urinary retention—occurs in <10% of AL cases and is easily misattributed to anxiety, cervical spondylosis, or idiopathic Parkinson’s disease, as in our patient. Several observations underscore the diagnostic challenge: orthostatic hypotension in AL is seldom corrected by volume expansion because it results from irreversible autonomic fiber destruction and vascular amyloid infiltration rather than hypovolemia. Nephrotic-range proteinuria in AL typically shows albumin <50% on urine protein electrophoresis, distinguishing it from primary glomerular disease. A serum κ/λ FLC ratio <0.26 (or >1.65) has >90% specificity for clonal light-chain disease, and our patient’s ratio of 0.26 was therefore strongly supportive of AL amyloidosis. These features emphasize the need to integrate tissue diagnosis, monoclonal protein studies, and clinical phenotyping to avoid diagnostic delays that continue to drive early mortality in AL amyloidosis.

Over the past decade, successive anti-plasma-cell advances, such as melphalan-prednisone, high-dose dexamethasone, thalidomide, bortezomib, and autologous stem cell transplantation, have incrementally improved survival in myeloma and related plasma-cell disorders (14–18). The most recent milestone was in 2021 when daratumumab, an anti-CD38 monoclonal antibody, was licensed for newly diagnosed AL amyloidosis in combination with cyclophosphamide, bortezomib, and dexamethasone (Dara-VCd) (19). In this patient, four cycles of Dara-VCd produced rapid hematological complete response and renal very-good-partial response, mirroring phase II/III data that show daratumumab-containing regimens double the depth and speed of response compared with VCd alone. CD38 targeting depletes clonal plasma cells and suppresses amyloidogenic light-chain production, whereas bortezomib amplifies endoplasmic reticulum stress and accelerates plasma cell apoptosis.

Supportive care is the cornerstone of management. Midodrine and droxidopa abolished recurrent syncope; without them, the patient’s orthostatic drops (>70 mmHg) would have carried a 30% annual risk of fall-related intracranial bleeding. Enoxaparin was mandatory while D-dimer peaked at 10.24 µg/mL, a surrogate for the pro-thrombotic state conferred by circulating SAP and endothelial amyloid. Autonomic-predominant disease still carries a median survival of only 8 months if untreated, chiefly due to arrhythmia and trauma. Even after hematological CR, autonomic recovery lagged by >12 months, highlighting two unmet needs: (i) nerve-specific biomarkers that quantify autonomic amyloid burden before irreversible axonal loss, and (ii) adjunctive neuroprotective agents to be administered concurrently with Dara-VCd.

AL amyloidosis is an insidious, protean disorder, and autonomic neuropathy predominance carries a high early mortality rate if untreated. In our case report, recognition at five months allowed prompt, aggressive therapy that converted a projected median survival of <12 months into sustained organ responses and preserved quality of life, underscoring that in the case of peripheral or autonomic neuropathy, nephrotic range proteinuria, hepatomegaly, and nondilated cardiomyopathy, the diagnosis should always be considered, even when serum or urine electrophoresis is negative.

## 5 Patient perspective

Although the patient did not complete the full course of targeted therapy combined with chemotherapy, her condition significantly improved, allowing her to manage daily activities and live independently.

## Data Availability

The original contributions presented in the study are included in the article/**Supplementary Material**. Further inquiries can be directed to the corresponding authors.
